# Isoquercitrin Suppresses Esophageal Squamous Cell Carcinoma (ESCC) by Inducing Excessive Autophagy and Promoting Apoptosis via the AKT/mTOR Signaling Pathway

**DOI:** 10.3390/antiox14060694

**Published:** 2025-06-08

**Authors:** Zhibin Liu, Ke Huang, Hai Huang, Eungyung Kim, Hyeonjin Kim, Chae Yeon Kim, Dong Joon Kim, Sang In Lee, Sangsik Kim, Do Yoon Kim, Kangdong Liu, Zae Young Ryoo, Mee-Hyun Lee, Lei Ma, Myoung Ok Kim

**Affiliations:** 1 Department of Animal Science and Biotechnology, Research Institute for Innovative Animal Science, Kyungpook National University, Sangju-si 37224, Gyeongsang buk-do, Republic of Korea; liuzhibin1124@naver.com (Z.L.); hk842767619@gmail.com (K.H.); jenus4@naver.com (H.K.); yyok1012@naver.com (C.Y.K.); silee78@knu.ac.kr (S.I.L.); 2 Henan International Joint Laboratory of TCM Syndrome and Prescription in Signaling, Traditional Chinese Medicine (Zhong Jing) School, Henan University of Chinese Medicine, Zhengzhou 450046, China; huanghai1227@126.com; 3 Department of Oral and Maxillofacial Surgery, School of Dentistry, University of Texas Health Science Cente at San Antonio, San Antonio, TX 78229, USA; wjddn5460@naver.com; 4 Department of Microbiology, College of Medicine, Dankook University, Cheonan 31116, Chungcheongnam-do, Republic of Korea; djkim@hci-cn.org; 5 Department of Energy Chemical Engineering, Kyungpook National University, Sangju-si 37224, Gyeongsang buk-do, Republic of Korea; sangsik@knu.ac.kr; 6 Convergence Research Center of Mechanical and Chemical Engineering, Kyungpook National University, 2559 Gyeongsang-daero, Sangju-si 37224, Gyeongsang buk-do, Republic of Korea; 7 Gyeongsangbukdo Livestock Research Institute, Yeongju 36052, Gyeongsang buk-do, Republic of Korea; kdy51311@korea.kr; 8 China–US (Henan) Hormel Cancer Institute, Zhengzhou 450008, China; kdliu@zzu.edu.cn; 9 BK21 FOUR KNU Creative BioResearch Group, School of Life Sciences, Kyungpook National University, Daegu 41566, Gyeongsang buk-do, Republic of Korea; jaewoong64@knu.ac.kr; 10 Korean Medicine Research Center for Bi-Wi Control Based Gut-Brain System Regulation, College of Korean Medicine, Dongshin University, Naju-si 58245, Jeollanam-do, Republic of Korea; mhlee@dsu.ac.kr

**Keywords:** esophageal squamous cell carcinoma, AKT/mTOR, anti-cancer, excessive autophagy, apoptosis

## Abstract

Esophageal squamous cell carcinoma (ESCC), one of the most frequent malignant tumors of the digestive system, is marked by a poor prognosis and high mortality rate. There is a critical need for effective therapeutic strategies with minimal side effects. Isoquercitrin (IQ) is a natural compound with potent antioxidant properties in cancer and cardiovascular diseases. However, its specific effects and mechanisms in ESCC remain largely unexplored. This study aims to investigate the effects of IQ in ESCC cells and elucidate the mechanisms underlying its therapeutic effects. Specifically, its impact on cell proliferation, colony formation, migration, and invasion was assessed using cell viability assay, morphology, transwell, and colony formation assays. The effects on apoptosis were evaluated by flow cytometry, while immunofluorescence (IF) staining and Western blotting were performed to confirm the underlying mechanisms. The in vivo anti-cancer effects of IQ were then evaluated using a xenograft tumor model. Our results demonstrate that IQ inhibits ESCC cell growth and colony formation while promoting its apoptosis by enhancing caspase activation and downregulating Bcl-2 expression. Furthermore, IQ suppresses cell migration by modulating the epithelial–mesenchymal transition-related proteins. Additionally, IQ induces excessive autophagy by promoting reactive oxygen species accumulation and inhibiting the AKT/mTOR signaling pathway. Importantly, IQ effectively reduces tumor growth in vivo, highlighting its potential as a therapeutic agent for ESCC.

## 1. Introduction

Esophageal squamous cell carcinoma (ESCC), one of the most frequent malignant tumors of the digestive system, is marked by a poor prognosis and high mortality rate [1,2]. Patients with esophageal cancer display a five-year survival rate of around 10%, while post-surgery survival rates range from 15% to 40% [3]. Despite significant efforts devoted to therapy development, the global incidence of esophageal cancer remains high. The major risk factors for the disease include the consumption of pickled vegetables, alcohol, and tobacco, which contribute to its high incidence in certain regions of Asia and Africa [4,5]. Currently, the three main treatment options for ESCC are radiotherapy, chemotherapy, and surgery. Although these treatments display high success rates, they frequently accompany serious side effects that negatively impact patients’ quality of life. Particularly, radiotherapy and chemotherapy often induce off-target toxicity to normal cells, leading to extensive collateral damage [6]. Consequently, there is a critical need for alternative therapeutic strategies that are both effective and less harmful.

Natural compounds have long been used in traditional medicine and are now widely recognized for their therapeutic effects, including antioxidant, anti-inflammatory, and anti-cancer properties [7,8,9]. Isoquercitrin (IQ), a flavonoid glycoside extracted from plants such as mango and rhubarb, is particularly recognized for its important pharmacological properties, including powerful antioxidant properties that help scavenge free radicals and reduce oxidative stress [10]. This property protects cells from reactive oxygen species (ROS), which are associated with various chronic diseases such as cancer and cardiovascular disease [4]. In addition, IQ displays significant anti-inflammatory effects by inhibiting the production of pro-inflammatory cytokines, which is beneficial in managing inflammatory diseases like arthritis [11]. Most notably, IQ exhibits anti-cancer properties by inhibiting the growth of cancer cells, as previously demonstrated for colorectal cancer and melanoma [12,13]. These properties underscore IQ’s vast potential as a novel therapeutic for various diseases.

Autophagy is highly adaptive and enables cells to cope with various forms of stress, such as protein and organelle damage and redox imbalance [14,15]. Consequently, autophagy displays a dual functionality in promoting both cell survival and death. The phosphatidylinositol 3-kinase (PI3K)/Akt/mammalian target of the rapamycin (mTOR) signaling pathway, a key regulator of autophagy, is involved in the initiation and progression of various tumor types [12,13,16], including ESCC. This presents a promising opportunity for therapeutic intervention [17]. It has been demonstrated that various natural compounds can modulate this pathway to inhibit tumor growth by inducing autophagy and apoptosis in cancer cells [18,19,20,21,22]. Despite such promising findings, the specific effects and mechanisms of IQ in ESCC remain largely unexplored.

Therefore, this study aims to investigate the effects of IQ in ESCC and elucidate the mechanisms underlying its therapeutic effects. We particularly focused on IQ’s role in modulating the AKT/mTOR pathway to induce autophagy and apoptosis. By elucidating the associated mechanisms, we aim to contribute to the development of effective and safe alternative therapeutic options for ESCC.

## 2. Materials and Methods

### 2.1. Ethical Statement

The Kyungpook National University (KNU) Institutional Review Board (KNU 2025-0005) approved the animal study protocol of this study.

### 2.2. Reagents and Antibodies

Various working concentrations of IQ (≥98% purity; Chengdu Must Bio-Technology Co., Chengdu, China) were prepared in dimethyl sulfoxide (DMSO). Cisplatin (CDDP) was generously provided by Dr. Yong-Gun Kim from the Department of Periodontology, Kyungpook National University School of Dentistry, Daegu, Republic of Korea. The primary antibodies against the following proteins were obtained from Santa Cruz Biotechnology Inc. (Dallas, TX, USA) and diluted to the specified ratio: vimentin (1:1000), E-cadherin (1:1000), N-cadherin (1:1000), and β-actin (1:1000). The primary antibodies against the following proteins were obtained from Cell Signaling Technology (Danvers, MA, USA) and diluted to the specified ratio: LC3A/B (1:1000), p62 (1:1000), PARP (1:1000), caspase-3 (1:1000), cleaved caspase-3 (1:1000), caspase-9 (1:1000), cleaved caspase-9 (1:1000), Bax (1:1000), Bcl-2 (1:1000), phospho-AKT (p-AKT; 1:1000), AKT (1:1000), phospho-mTOR (p-mTOR; 1:1000), and mTOR (1:1000).

### 2.3. Cell Culture

The ESCC cell lines KYSE-510, KYSE-450, and KYSE-70 were obtained from the China-US (Henan) Hormel Cancer Institute (Zhengzhou, China). The cells were cultured in Roswell Park Memorial Institute 1640 medium (RPMI-1640; Gibco™, Grand Island, NY, USA) supplemented with 10% fetal bovine serum (FBS; GenDEPOT, Katy, TX, USA) and 1% penicillin-streptomycin (PS; Gibco™). Primary human normal fibroblasts (HGnFs) were obtained from tissue samples collected from 3 healthy donors under the age of 35, with no systemic disease. Tissue biopsies approximately 1 mm in size were used to isolate fibroblasts, which were then cultured in Dulbecco’s Modified Eagle Medium (DMEM; Gibco™, Waltham, MA, USA) containing 10% fetal bovine serum (FBS; GenDEPOT, Katy, TX, USA) and 1% penicillin–streptomycin solution (Gibco™, Waltham, MA, USA). All cell lines were maintained at 37 °C in a humidified atmosphere with 5% CO_2_.

### 2.4. Cell Viability Assay

Cell viability was assessed using the Cell Counting Kit-8 (CCK-8; Dojindo, Mashiki, Japan) assay. Briefly, cells were seeded in 96-well plates at a density of 2 × 10^3^ cells per well. IQ was treated to cells at final concentrations of 0 µM, 200 µM, 400 µM, and 800 µM. Cell viability was measured at 0, 24, 48, 72, and 96 h after treatment by adding 10 µL of CCK-8 solution to each well and incubating for 3 h. The absorbance was read at 450 nm using a microplate reader (Thermo Fisher Scientific, Waltham, MA, USA).

### 2.5. Cell Morphology Analysis

To analyze cellular morphology, cells were seeded in 6-well plates at a density of 2 × 10^5^ cells per well and treated with IQ at the same concentrations and durations tested in the viability assay. Cell morphology was observed and photographed using an inverted light microscope.

### 2.6. Colony Formation Assay

Cells were seeded in 6-well plates at a density of 1 × 10^3^ cells per well and treated with varying concentrations of IQ for 10 days. Colonies were then fixed with 4% formaldehyde for 15 min and stained with 0.1% crystal violet for 20 min. The colony formation degrees were analyzed using the ImageJ software (version 1.53).

### 2.7. Cell Migration and Invasion Assay

For the invasion assay, the upper chamber was prepared in advance. Matrigel (Corning Costar, Lowell, MA, USA) was diluted at a 1:9 ratio with serum-free DMEM and evenly applied to the bottom of the upper chamber at a volume of 100 μL per well. After application, the upper chamber was placed into the corresponding lower chamber and incubated at 37 °C overnight. Once the Matrigel solidified, the culture plate was removed. No such preparation was required for the migration assay. The subsequent procedures for the migration and invasion assays were identical. Cells were seeded in the upper chamber of the transwell inserts at a density of 2 × 10^4^ cells per well in serum-free medium, while the lower chamber contained medium supplemented with 10% FBS. After 48 h, cells that had migrated to the lower surface of the membrane were fixed with 4% formaldehyde, stained with crystal violet, and observed under a microscope.

### 2.8. ROS Assay

To detect ROS levels in cells, 2 × 10^4^ cells were seeded in a 4-well culture slide (SPL Life Sciences, Pocheon-si, Republic of Korea) and incubated at 37 °C for 48 h after treating the corresponding drugs. The cells were then stained using the DCFDA/H2DCFDA-Cellular ROS Assay Kit (Abcam, Cambridge, UK) for 45 min at 37 °C. Subsequently, the cells were washed thrice with phosphate-buffered saline (PBS). Finally, images were captured using a fluorescence microscope, and differences in fluorescence intensity between the samples were analyzed.

### 2.9. Cell Apoptosis Analysis

The degree of apoptosis was measured by flow cytometry of the treated cells. Cells were seeded in 6-well plates at a density of 2 × 10^5^ cells per well, treated with IQ for 48 h, and then harvested and washed twice with cold PBS. The cells were resuspended in a binding buffer and stained with Annexin V-FITC (Invitrogen, Thermo Fisher Scientific, Waltham, MA, USA) and propidium iodide (PI) according to the manufacturer’s instructions. The stained cells were analyzed using the FACS Verse Flow Cytometry system (BD Science, Milpitas, CA, USA).

### 2.10. Western Blot Analysis

Cells were lysed in RIPA buffer containing protease and phosphatase inhibitors. Protein concentrations were determined using the BCA assay. Equal amounts of protein extracts were separated by SDS-PAGE and transferred to PVDF membranes. The membranes were then blocked with 5% non-fat milk in TBST and incubated overnight at 4 °C with primary antibodies against vimentin, N-cadherin, E-cadherin, LC3A/B, p62, caspase-3, cleaved caspase-3, caspase-9, cleaved caspase-9, PARP, p-AKT (Ser473), p-mTOR (Ser2448), AKT, and mTOR. After washing, the membranes were incubated with HRP-conjugated secondary antibodies for 1 h at room temperature. Bands were visualized using the Thermo Scientific SuperSignal™ West Pico PLUS (Thermo Fisher Scientific) chemiluminescent substrate and imaged with the ImageQuant LAS 500 System (Cytiva, New York, NY, USA).

### 2.11. Immunofluorescence (IF) Staining

To check the protein expression levels in cells, 2 × 10^4^ cells were seeded in a 4-well culture slide (SPL Life Sciences) and incubated at 37 °C for 48 h after treating the corresponding drugs. The cells were fixed with 4% paraformaldehyde for 10 min and blocked with 10% normal goat serum (NGS) for 2 h after permeabilization with 0.1% TrintonX-100 was used for 10  min. After washing with 1% PBST thrice, the cells were incubated with the primary antibody against LC3A/B and p62 at 4 °C overnight and then incubated with the appropriate fluorescent secondary antibody at room temperature for 2  h. The cell nuclei were counter-stained with 4′, 6-diamidino-2-phenylindole (DAPI) and analyzed by laser scanning confocal microscopy.

### 2.12. Cell-Derived Xenograft (CDX) Mouse Tumor Model

Animal experiments were approved by the Ethics Committee of Kyungpook National University (Daegu, Republic of Korea). KYSE-510 cells were inoculated in BALB/c-nude mice (4 to 5 weeks of age; RaonBio, Yongin-si, Republic of Korea) to establish a CDX mouse tumor model. A total of 5 × 10^6^ KYSE-510 cells were subcutaneously injected into the right flank of each mouse. Once the tumors reached an average volume of 80–120 mm^3^, the mice were randomly divided into three groups (8 mice per group) and treated with either the vehicle or IQ (10 mg/kg and 20 mg/kg) twice a week. The mouse body weights were recorded. The tumor length and width were measured using a caliper, and the tumor volume was calculated as follows: length × width^2^ × 0.52. Finally, the mice were euthanized, and the tumors, kidneys, liver, lungs, and spleen were harvested for further analysis by Western blot, hematoxylin-eosin (H&E) staining, and immunohistochemistry (IHC).

### 2.13. IHC Analysis

Paraffin-embedded sections (5 μm) were prepared for IHC analysis. Tumor tissue samples were dewaxed, hydrated, and permeabilized with 0.5% Triton X-100 in PBS for 10 min. Then, the sections were blocked with 10% NGS and incubated with the primary antibodies against Ki-67, p-AKT, and p-mTOR at 4 °C overnight. Subsequently, the sections were washed thrice with PBS and incubated with the appropriate secondary antibodies. According to the manufacturer’s instructions, 3,3′-diaminobenzidine (DAB) staining was conducted to visualize the protein targets. Finally, the sections were counter-stained with H&E for 5 min and photographed using a microscope.

### 2.14. Statistical Analysis

All experiments were performed in triplicate, and data are expressed as mean ± standard deviation (SD). Statistical analysis was performed using GraphPad Prism 9 (San Diego, CA, USA) and the SPSS software package (SPSS Statistics v.23.0; IBM, Chicago, IL, USA). Student’s *t*-test was used for analysis. A *p*-value of <0.05 was considered statistically significant.

## 3. Results

### 3.1. IQ Inhibits ESCC Cell Growth

**Figure 1:**
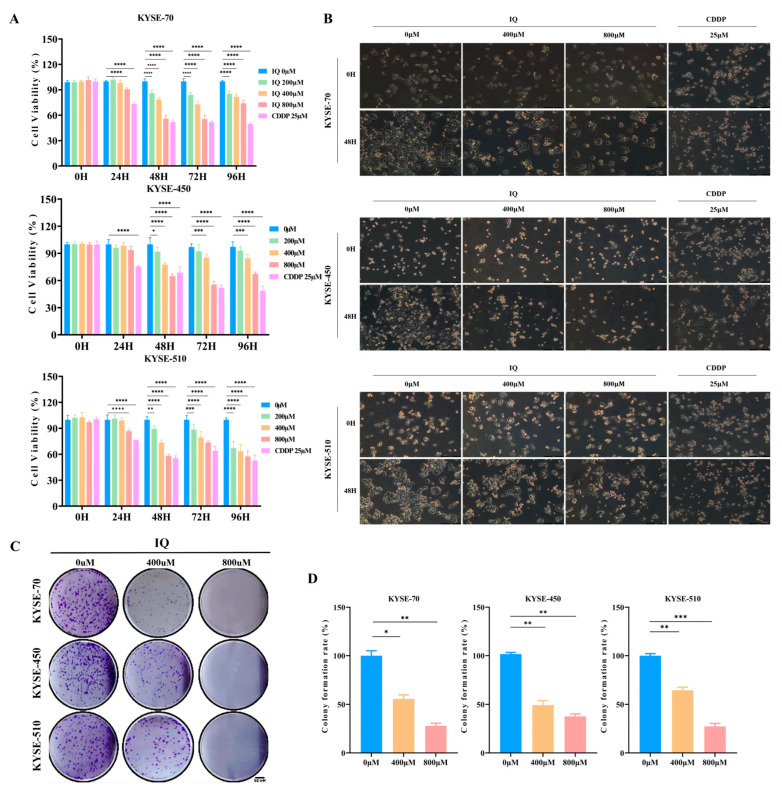
IQ inhibits ESCC viability and colony formation. (**A**) Viability of KYSE-70, KYSE-450, and KYSE-510 cells treated with varying concentrations of IQ (0, 200, 400, and 800 µM) and CDDP (25 µM) for 0, 24, 48, 72, and 96 h. Cell viability was measured using the CCK-8 assay. (**B**) Morphology of KYSE-70, KYSE-450, and KYSE-510 cells treated with CDDP (25 µM) and IQ at 0, 400, and 800 µM for 48 h, observed under light microscopy (Scale bar = 316.5 µM). (**C**) Colony formation of KYSE-70, KYSE-450, and KYSE-510 cells treated with 0, 400, and 800 µM IQ for 14 days. The colonies were stained with crystal violet for quantification. (**D**) Quantified colonies of KYSE-70, KYSE-450, and KYSE-510 cells. The colony counts were normalized to the untreated control. * *p* < 0.05, ** *p* < 0.01, *** *p* < 0.001, and **** *p* < 0.0001 compared to the control group.

Based on the results of IQ treatment in human normal fibroblast, a non-toxic concentration was identified using the CCK-8 assay and applied in subsequent experiments to ensure safety and selectivity (Figure S1). IQ showed a dose-dependent inhibition of ESCC cell growth across all tested cell lines (KYSE-70, KYSE-450, and KYSE-510) and IQ concentrations (200 µM, 400 µM, and 800 µM), leading to significantly lower cell viability compared to the untreated control group. Growth inhibition was most pronounced at 800 µM IQ and with longer exposure times of 48 to 96 h. Meanwhile, the positive control cisplatin (CDDP, 25 μM) significantly reduced cell viability at 48, 72, and 96 h, demonstrating strong cytotoxic effects (Figure 1A). Morphological analysis revealed a dose-dependent decrease in the number of ESCC cells following IQ and CDDP treatment. Furthermore, compared with the control group, both CDDP- and IQ-treated cells exhibited typical morphological features of apoptosis or cell death, including cell shrinkage, reduced cell density, and partial detachment or fragmentation (Figure 1B). Overall, the results indicate that the anti-tumor efficacy of IQ is comparable to that of the standard chemotherapeutic agent CDDP and that its effects are both time- and dose-dependent. These alterations, along with signs of cell shrinkage and nuclear condensation, suggested that IQ treatment induces apoptosis and excessive autophagy in ESCC cells (Figure S2). The colony formation assay was performed to assess the long-term clonogenic potential of ESCC cells treated with IQ. After 10 days of treatment, a marked reduction in the number and size of colonies was observed in all IQ-treated groups compared to in the control group. The inhibition was dose-dependent, with the most substantial reduction observed at 800 µM IQ. The colony formation assay results indicated that IQ significantly inhibits the clonogenic potential of ESCC cells (Figure 1C,D).

### 3.2. IQ Suppresses the Migration and Invasion of ESCC Cells

**Figure 2:**
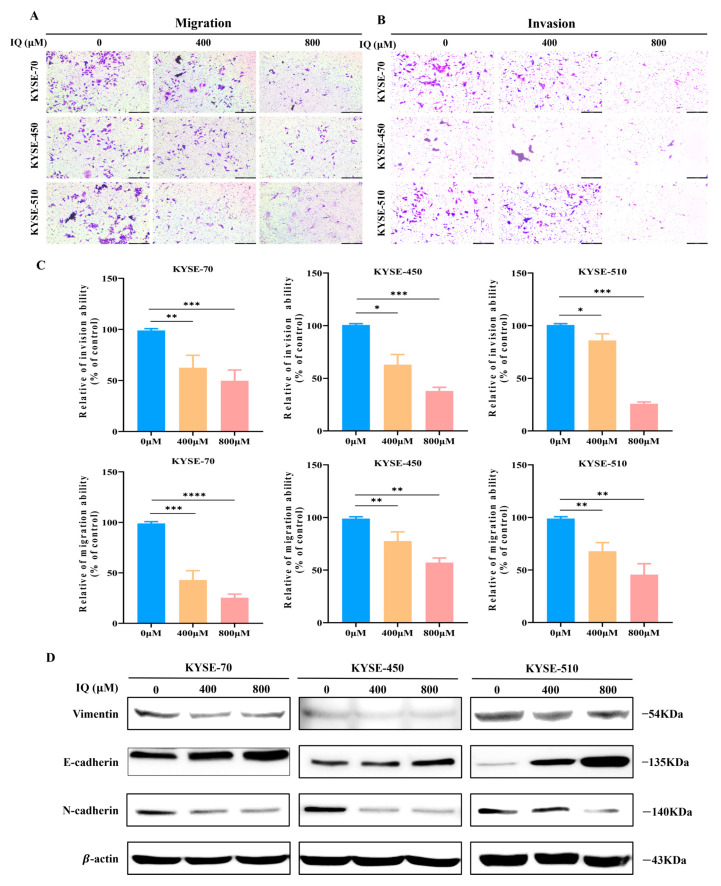
IQ suppresses ESCC migration and invasion by modulating the expression of EMT-related proteins. (**A**) Representative images of transwell migration of KYSE-70, KYSE-450, and KYSE-510 cells treated with IQ at 0, 400, and 800 µM for 24 h. The migrated cells were stained with crystal violet and observed under a light microscope (Scale bar = 316.5 µM). (**B**) Representative images of ESCC cell invasion through the Matrigel-coated membrane following IQ treatment. IQ inhibited cell invasion in a dose-dependent manner (Scale bar = 316.5 µM). (**C**) Quantification of the cell area for each treatment condition’s migration and invasion of KYSE-70, KYSE-450, and KYSE-510 cells. (**D**) Western blot analysis of EMT-related proteins in KYSE-70, KYSE-450, and KYSE-510 cells treated with IQ at 0, 400, and 800 µM for 48 h. Protein expression levels of E-cadherin (epithelial marker), N-cadherin, and vimentin (mesenchymal markers) were assessed. β-actin was used as the loading control. * *p* < 0.05, ** *p* < 0.01, *** *p* < 0.001, and **** *p* < 0.0001 compared to the control group.

The metastatic potential of IQ-treated ESCC cells was assessed by transwell migration and invasion assays. IQ significantly inhibited the migration and invasion of ESCC cells in a dose-dependent manner, with the greatest reduction observed at 800 µM (Figure 2A–C). This suggested that IQ impairs the migratory and potentially metastatic capabilities of ESCC cells. To evaluate the effects of IQ on the epithelial–mesenchymal transition (EMT) process in ESCC cells, Western blot analysis was conducted for the EMT-related markers. The expression of E-cadherin was upregulated, while N-cadherin and vimentin were downregulated in IQ-treated ESCC cells compared to the untreated control group (Figure 2D). Collectively, it was demonstrated that IQ induces anti-cancer effects by inhibiting the EMT process in ESCC cells, thereby reducing their invasiveness and metastatic potential.

### 3.3. IQ Induces ROS Generation and Promotes Autophagy in ESCC Cells

**Figure 3:**
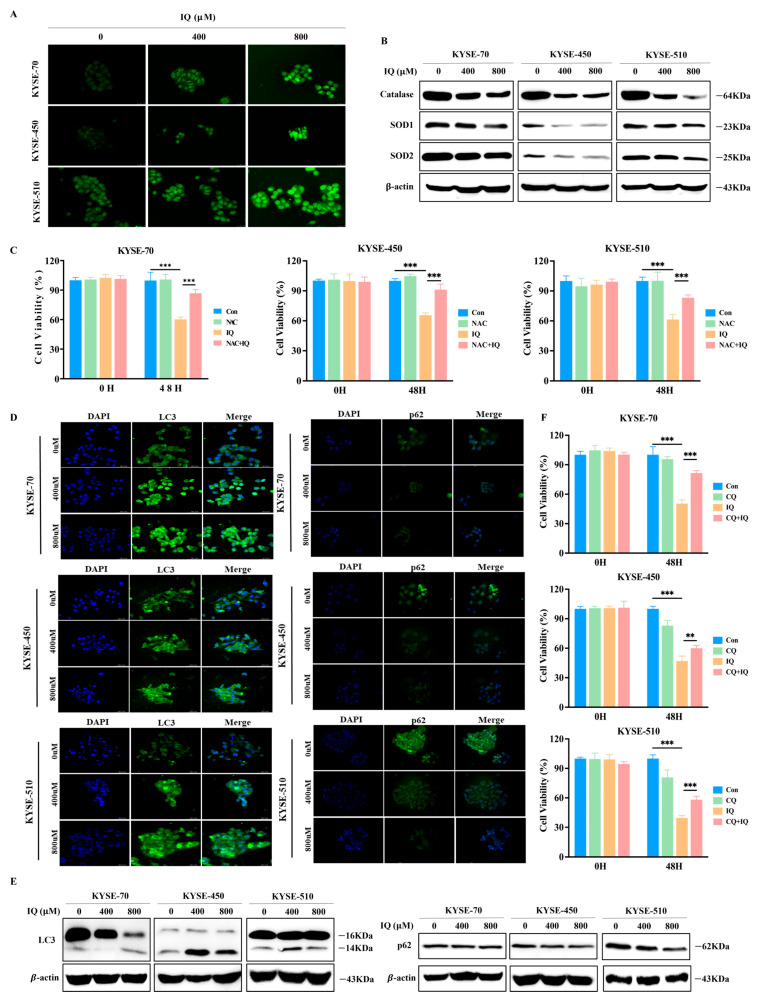
IQ induces ROS generation and promotes autophagy in ESCC cells. (**A**) Representative images of ROS in KYSE-70, KYSE-450, and KYSE-510 cells treated with IQ at 0, 400, and 800 µM for 48 h. The cells were stained with DCFH-DA (green fluorescence) and imaged by fluorescence microscopy (scale bar = 25 µM). (**B**) Western blot analysis of the antioxidant proteins catalase, SOD1, and SOD2 in KYSE-70, KYSE-450, and KYSE-510 cells after treatment with 0, 400, and 800 µM IQ. (**C**) Viability of ESCC cells cotreated with IQ and the autophagy inhibitor NAC (500 µM). (**D**) IF staining of LC3 and p62 in KYSE-70, KYSE-450, and KYSE-510 cells treated with IQ at 0, 400, and 800 µM for 48 h. Green fluorescence indicates LC3 puncta, while blue fluorescence represents DAPI-stained nuclei (Scale bar = 161.2 µM). (**E**) Western blot analysis of the autophagy-related proteins LC3-II/I and p62 in KYSE-70, KYSE-450, and KYSE-510 cells after treatment with 0, 400, and 800 µM IQ. (**F**) Viability of ESCC cells cotreated with IQ and the autophagy inhibitor CQ (20 µM). Data are presented as mean ± SD with the following significance levels compared to the control group: ** *p* < 0.01, *** *p* < 0.001.

We observed that the morphology of IQ-treated ESCC cells resembled that of autophagic cells (Figure S2). Previous studies have shown that ROS are involved in signal transduction and regulation in cells [14,15], leading to a direct impact on cell death and autophagy [16,17,18,19]. Therefore, in this study, we investigated the changes in ROS levels in IQ-treated ESCC cells. We observed that IQ treatment increases cellular ROS levels in a dose-dependent manner (Figure 3A). Furthermore, Western blot analysis showed that the expression levels of antioxidant proteins, such as catalase, SOD1, and SOD2, were decreased in IQ-treated ESCC cells (Figure 3B). These findings indicated that IQ treatment induces ROS generation in ESCC cells. Our CCK-8 assay results showed that NAC alone had little effect on ESCC cell viability, while IQ alone significantly reduced cell viability, as expected. Importantly, cotreatment with ROS inhibitor N-acetylcysteine (NAC) and IQ led to a partial restoration of cell viability, suggesting that scavenging ROS can attenuate the cytotoxic effects of IQ. These findings provide direct functional evidence that ROS play a main role in IQ-induced cell death (Figure 3C).

Next, we performed IF staining to detect the levels of the autophagy protein LC3 in the cytoplasm and the autophagosome-lysosome fusion protein p62 [20,21]. The results showed a dose-dependent increase in LC3 expression and a decrease in p62 expression across all three IQ-treated ESCC cell lines (Figure 3D). Additionally, Western blot analysis confirmed the consistent expression patterns of these two proteins (Figure 3E).

We then sought to investigate the mechanism underlying IQ-induced autophagy using the autophagy inhibitor chloroquine (CQ). It was shown that the viability of ESCC cells was significantly reduced with IQ alone but was partially restored when IQ and CQ were treated together (Figure 3F). These findings confirmed that IQ promotes autophagy in ESCC cells by modulating intracellular ROS levels.

### 3.4. IQ Induces Apoptosis in ESCC Cells by Modulating the AKT/mTOR Signaling Pathway

**Figure 4:**
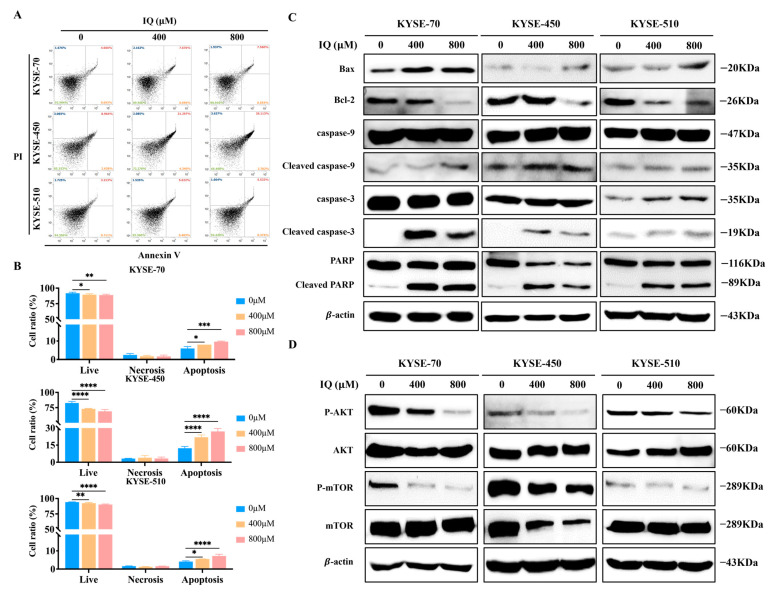
IQ induces apoptosis in ESCC cells by modulating the AKT/mTOR signaling pathway. (**A**) Flow cytometry of KYSE-70, KYSE-450, and KYSE-510 cells treated with IQ at 0, 400, and 800 µM for 48 h. Annexin V-FITC/PI staining was used to detect early and late apoptotic cells. (**B**) Quantification of apoptotic KYSE-70, KYSE-450, and KYSE-510 cells. (**C**) Western blot analysis of apoptosis-related proteins in KYSE-70, KYSE-450, and KYSE-510 cells treated with IQ at 0, 400, and 800 µM. (**D**) Western blot analysis of key proteins in the AKT/mTOR signaling pathway, including AKT, p-AKT, mTOR, and p-mTOR, in KYSE-70, KYSE-450, and KYSE-510 cells treated with IQ at 0, 400, and 800 µM for 48 h. * *p* < 0.05, ** *p* < 0.01, *** *p* < 0.001, and **** *p* < 0.0001 compared to the control group.

Most cancer therapies induce cytotoxic stress on cancer cells, leading to their death [22]. Previous studies have shown that excessive autophagy can induce autophagic cell death [23,24,25]. IQ-induced ESCC apoptosis was further investigated by analyzing the expression of apoptosis-related proteins via flow cytometry and Western blot. Flow cytometry indicated a dose-dependent increase in both early and late apoptosis in IQ-treated ESCC cells (Figure 4A,B). Western blot analysis corroborated these findings, showing elevated levels of Bax, cleaved caspase-9, cleaved caspase-3, and cleaved PARP, while Bcl-2 expression was decreased (Figure 4C). These results suggested that IQ-induced apoptosis in ESCC cells is indeed associated with apoptosis-related protein regulation, which activates the caspase-dependent apoptotic pathway.

Previous studies have shown that targeting the AKT/mTOR signaling pathway can induce cell autophagy and death in gastric and lung cancers, offering potential therapeutic strategies [26,27,28]. To further explore the potential function and molecular mechanism of IQ in ESCC, the impact on the AKT/mTOR signaling pathway proteins was assessed by Western blot analysis. IQ treatment resulted in the decreased phosphorylation of AKT and mTOR (Figure 4D), indicating the inhibition of the AKT/mTOR pathway. These results indicated that IQ inhibits the AKT/mTOR signaling pathway to induce excessive autophagy in ESCC cells, thereby promoting apoptosis and suppressing growth.

### 3.5. IQ Suppresses the Growth of ESCC Tumors In Vivo

**Figure 5:**
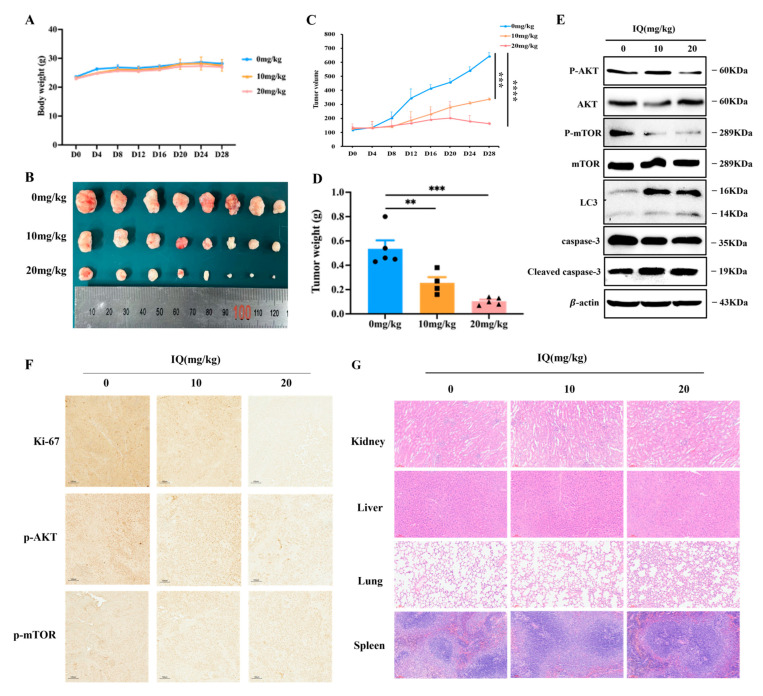
IQ suppresses the growth of ESCC tumors and modulates their characteristics in vivo. (**A**) Body weights of mice treated with IQ at doses of 0, 10, and 20 mg/kg over 28 days. (**B**) Representative images of tumor tissues excised from mice after 28 days of treatment with IQ at 0, 10, and 20 mg/kg. (**C**) Tumor growth curve showing average tumor volumes of the groups treated with IQ at 0, 10, and 20 mg/kg over 28 days. (**D**) Tumor weights after treatment with IQ at different doses. (**E**) Western blot analysis of key signaling proteins (p-AKT, AKT, p-mTOR, and mTOR) and the autophagy marker LC3-II in tumor tissues. (**F**) IHC analysis of tumor tissues for signaling proteins and Ki-67 (Scale bar = 80 µM). (**G**) H&E staining of tissues harvested from the control and IQ-treated groups. Scale bar = 100 µM. ** *p* < 0.01, *** *p* < 0.001, **** *p* < 0.0001.

To investigate whether IQ suppresses tumor growth in vivo, KYSE-510 tumors were established in mice. IQ administration significantly inhibited the growth of the tumors compared to the control group, as evidenced by reduced tumor volume and weight. Meanwhile, the body weight of the IQ-treated group remained comparable to that of the control group, confirming IQ’s safety (Figure 5A–D).

Moreover, p-AKT and p-mTOR expressions were significantly decreased in the tumors of the IQ-treated group, while LC3 and cleaved caspase-3 were markedly increased (Figure 5E). IHC analysis indicated the proliferation marker protein Ki-67, along with p-AKT and p-mTOR, was significantly reduced in the treatment group compared to in the control group (Figure 5F). H&E staining indicated that the morphology of kidneys, livers, lungs, and spleens of the treatment group remained normal, with no sign of cancer metastases to distant organs (Figure 5G). These findings collectively suggested that IQ exerts a dose-dependent anti-tumor effect both in vitro and in vivo by inhibiting the AKT/mTOR signaling pathway.

## 4. Discussion

This study highlights IQ, a natural flavonoid compound, as a potent anti-tumor agent against ESCC. Natural compounds have garnered significant attention for their therapeutic potential and reduced side effects compared to conventional chemotherapy and radiotherapy [29,30]. In particular, IQ’s antioxidant, anti-inflammatory, and anti-cancer properties are widely recognized [10,11,31,32].

It was demonstrated that IQ promotes autophagy-mediated cell death in a dose-dependent manner by inhibiting the AKT/mTOR pathway, consistent with previous studies on autophagy-inducing compounds in malignancies [33,34,35,36]. Furthermore, it was demonstrated that IQ-induced ROS generation is closely associated with the regulation of autophagy and apoptosis, further emphasizing the interconnected roles of these pathways in cancer cell death. Interestingly, while elevated ROS levels typically trigger the upregulation of antioxidant defenses, our study revealed a downregulation of catalase, SOD1, and SOD2 following IQ treatment. This inverse relationship suggests that IQ induces a level of oxidative stress that exceeds the threshold for cellular compensation, thereby suppressing or exhausting the antioxidant response. Similar observations have been reported for other flavonoids that interfere with Nrf2-mediated antioxidant pathways [37,38]. The collapse of this defense system may further amplify oxidative damage and contribute to the induction of autophagy and apoptosis in ESCC cells.

By inducing both apoptosis and autophagy in ESCC cells, IQ has the ability to exert strong anti-cancer effects. IQ-induced apoptosis was confirmed by upregulated levels of Bax and cleaved caspase-3, along with downregulated Bcl-2, as demonstrated through flow cytometry and Western blot analyses (Figure 4A–C). IQ-induced autophagy was evidenced by a dose-dependent increase in LC3 and a decrease in p62 expression, with the autophagy inhibitor CQ partially alleviating IQ-induced cytotoxicity (Figure 3). These findings indicate that IQ treatment induces excessive autophagy, leading to cell death. This is consistent with previous studies suggesting that clearing damaged organelles or promoting cell death under dysregulated conditions can be effective strategies for cancer therapy [23,39,40]. Additionally, ROS production is recognized as an upstream regulator of autophagy and apoptosis [16,41,42], in which elevated ROS levels are prevalent. In this regard, the observed IQ-induced ROS generation and subsequent inhibition of ESCC cell migration and invasion highlight its therapeutic potential (Figure 2).

Furthermore, IQ treatment was shown to inhibit the phosphorylation of AKT and mTOR, which play a key role in regulating cell survival, proliferation, and autophagy [43,44,45]. The AKT/mTOR pathway is a recognized driver of tumor growth and survival in ESCC and other malignancies [46,47]. Interestingly, a recent study reported that IQ exerts anti-cancer effects in gastric cancer cells by inducing endoplasmic reticulum (ER) stress and promoting immunogenic cell death [48]. While this study highlights a distinct mechanism of IQ action in gastric cancer, our findings reveal that in esophageal squamous cell carcinoma (ESCC), IQ induces excessive autophagy and apoptosis primarily through inhibition of the AKT/mTOR signaling pathway and elevation of intracellular ROS levels. These differences suggest that IQ may act via cancer-type-specific mechanisms, possibly influenced by the molecular context of each tumor type. This further underscores the novelty of our study in elucidating IQ’s anti-tumor effects in ESCC, which to our knowledge has not been previously reported. In this study, these effects of IQ were validated in vivo, as evidenced by reduced tumor growth, tumor volume, and p-AKT and p-mTOR expression, along with increased levels of LC3 and cleaved caspase-3 (Figure 5). Therefore, IQ’s ability to downregulate phosphorylated AKT and mTOR highlights its therapeutic potential as an agent that both inhibits tumor proliferation and induces cell death through autophagy. The in vivo results obtained from the xenograft mouse model further corroborated our in vitro findings. IQ treatment led to a significant reduction in tumor volume and weight, without affecting body weight, indicating both efficacy and tolerability. Mechanistically, the decreased expression of phosphorylated AKT and mTOR, as well as the increased levels of LC3 and cleaved caspase-3 observed in tumor tissues, mirror the molecular effects identified in IQ-treated ESCC cell lines. This parallel between in vitro and in vivo results strongly supports the conclusion that IQ exerts its anti-tumor activity by inducing excessive autophagy and apoptosis through inhibition of the AKT/mTOR pathway. The consistency across both experimental systems reinforces the translational relevance of our findings and highlights the therapeutic potential of IQ for ESCC.

**Figure 6:**
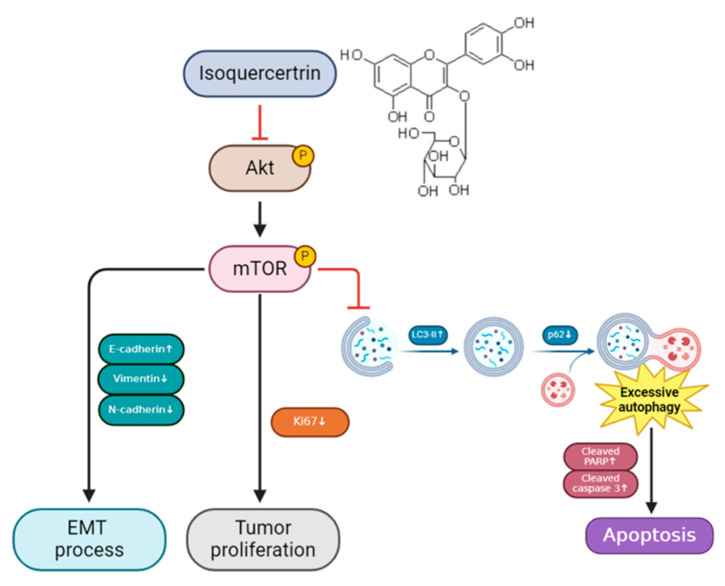
IQ inhibits esophageal squamous cell carcinoma progression by inducing excessive autophagy through the regulation of the AKT/mTOR pathway. The upward and downward arrows represent increased and decreased protein expression levels, respectively. (Created in BioRender. HUANG, K. (2025) https://BioRender.com/y53f756 accessed on 6 March 2025).

IQ inhibits the phosphorylation of AKT and mTOR in the AKT/mTOR signaling pathway. This results in the induction of autophagy, as evidenced by increased LC3-II expression and decreased levels of p62. Excessive autophagy contributes to tumor cell apoptosis, as indicated by the upregulation of cleaved PARP and cleaved caspase-3. Furthermore, IQ suppresses EMT by upregulating E-cadherin (epithelial marker) and downregulating vimentin and N-cadherin (mesenchymal markers). This leads to reduced tumor invasiveness. IQ also inhibits tumor proliferation, as reflected by the decreased Ki67 expression. Collectively, these effects suggest that IQ exerts its anti-tumor effects in ESCC by modulating autophagy, apoptosis, EMT, and tumor proliferation (Figure 6).

## 5. Conclusions

Our findings demonstrate that IQ treatment effectively inhibits ESCC growth, migration, and invasion both in vitro and in vivo. IQ treatment was found to enhance LC3 expression while reducing p62, thereby inducing excessive autophagy and promoting apoptosis in ESCC cells via the AKT/mTOR signaling pathway. These results suggest IQ’s significant potential as a therapeutic agent for ESCC.

## Data Availability

Data is contained within the article and Supplementary Materials.

## Supplementary Materials

The following supporting information can be downloaded at: https://www.mdpi.com/article/10.3390/antiox14060694/s1, Figure S1: To evaluate the selectivity of IQ, we assessed its cytotoxicity in human normal fibroblast (HGnF) using the CCK-8 assay. As shown in Figure S1, IQ exhibited minimal toxicity to HGNF cells even at high concentrations (up to 1600 μM). The IC_50_ values for 24 h and 48 h treatments were 142.08 mM and 35.73 mM, respectively, which are significantly higher than the concentrations that inhibited ESCC cell viability. These results indicate that IQ exerts selective cytotoxicity toward tumor cells while sparing normal cells; Figure S2: Morphology of IQ-treated ESCC cells. Representative images show a dose-dependent decrease in ESCC cell number and significant morphological changes following IQ treatment. The cells exhibit rounding, membrane blebbing, and detachment, along with characteristics of apoptosis, such as cell shrinkage and nuclear condensation. These observations suggest that IQ treatment induces apoptosis and excessive autophagy in ESCC cells.
