# Supplementary material for: Isoquercitrin Suppresses Esophageal Squamous Cell Carcinoma (ESCC) by Inducing Excessive Autophagy and Promoting Apoptosis via the AKT/mTOR Signaling Pathway
Source: Antioxidants (Basel). 2025 Jun 8;14(6):694. doi: 10.3390/antiox14060694 (PMC12189870; doi:10.3390/antiox14060694)
Supplement: Supplementary file 1 [file antioxidants-14-00694-s001.zip › antioxidants-3601664-supplementary.pdf]

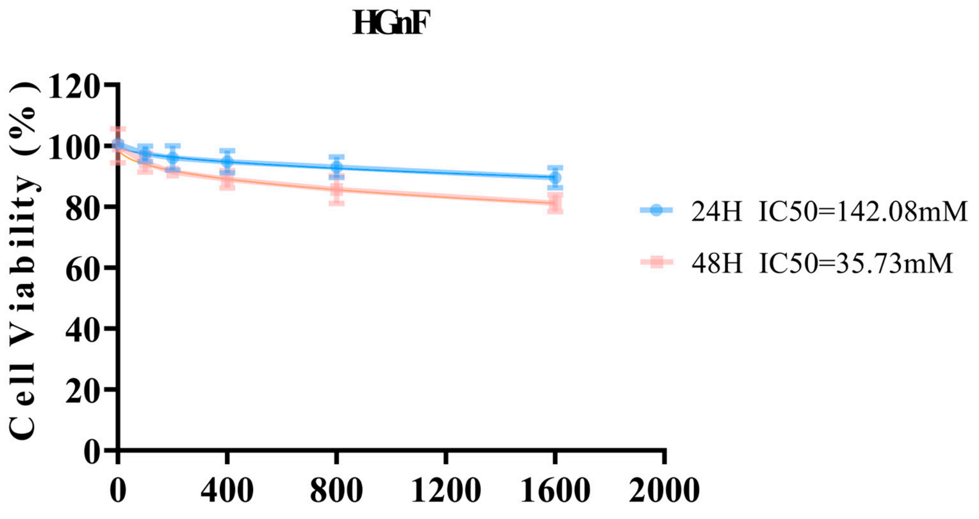

Figure S1. To evaluate the selectivity of IQ, we assessed its cytotoxicity in human normal fibroblast (HGnF) using the CCK-8 assay. As shown in Figure S1, IQ exhibited minimal toxicity to HGNF cells even at high concentrations (up to 1600 μM). The IC_{50} values for 24 h and 48 h treatments were 142.08 mM and 35.73 mM, respectively, which are significantly higher than the concentrations that inhibited ESCC cell viability. These results indicate that IQ exerts selective cytotoxicity toward tumor cells while sparing normal cells.

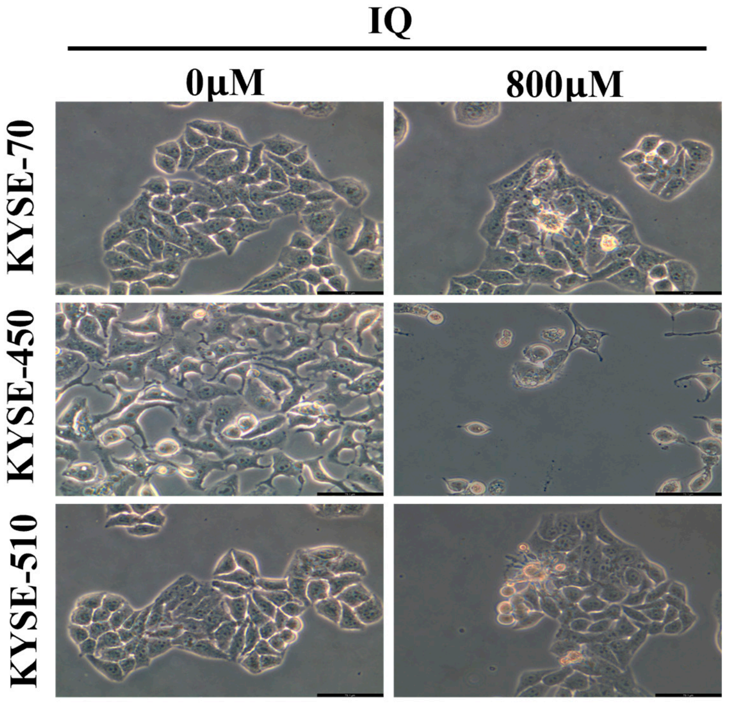

Figure S2. Morphology of IQ-treated ESCC cells. Representative images show a dose-dependent decrease in ESCC cell number and significant morphological changes following IQ treatment. The cells exhibit rounding, membrane blebbing, and detachment, along with characteristics of apoptosis, such as cell shrinkage and nuclear condensation. These observations suggest that IQ treatment induces apoptosis and excessive autophagy in ESCC cells (Scale bar = 79.1 μM).
